# Myocardial synchronized exercise and prognosis in patients with heart failure with preserved ejection fraction assessed by two-dimensional ultrasound speckle tracking stratified strain imaging

**DOI:** 10.1097/MD.0000000000041274

**Published:** 2025-01-17

**Authors:** Jingwan Chen, Xidan Wang, Fuhua Chen, Wenchao Weng, Biao Tang, Yibo Zhou

**Affiliations:** a Department of Ultrasound, JinHua Municipal Central Hospital, Jinhua, Zhejiang, China; b Department of Cardiology, JinHua Municipal Central Hospital, Jinhua, Zhejiang, China.

**Keywords:** heart failure with preserved ejection fraction, myocardial synchronized motion, overall longitudinal strain of the left ventricle, peak strain dispersion, stratified strain imaging, ultrasound speckle tracking

## Abstract

To evaluate myocardial synchronized exercise and clinical prognosis in patients with heart failure preserved ejection fraction (HFpEF), we utilized two-dimensional speckle tracking (2D-STI) stratified strain imaging. We retrospectively summarized 146 patients diagnosed with HFpEF in our hospital from January 2022 to January 2023. 2D-STI combined with stratified strain imaging was used to measure the overall left ventricular global longitudinal strain (LVGLS), the sub-endocardium, mid-myocardium, sub-epicardium LS of the left ventricle, as well as the basal, intermediate, and apical LS, the peak strain dispersion (PSD) and the transmural pressure difference, the postsystolic shortening (PSS), and early systolic lengthening. They were categorized into adverse and better prognosis groups based on major adverse cardiac events (MACE). MACE occurred in a total of 25 of 146 patients (17.12%). Compared with the better group (*P* < .05), there were significant differences in ages, incidences of myocardial infarction, pre-admission plasma brain natriuretic peptide levels, LVGLS, sub-endocardium and sub-epicardium LS, PSD, and PSS values in the adverse group. Compared to pretreatment in the better group at 1-month follow-up, LVGLS, sub-endocardium, mid-level, sub-epicardium LS, PSD, and PSS values improved significantly (*P* < .05), but the adverse group did not (*P* > .05). Multivariate Cox regression demonstrated that pretreatment LVGLS (HR = 1.362, 95% CI = 1.026–1.809, *P* = .033), sub-epicardium LS (HR = 1.669, 95% CI = 1.068–2.609, *P* = .025), and PSD values (HR = 1.075, 95% CI = 1.014–1.140, *P* = .015) were important predictors of the occurrence of MACE in patients with HFpEF. The receiver operating curves manifested that the area under the curve of pretreatment LVGLS, sub-epicardium LS, and PSD values for predicting the occurrence of MACE were 0.812 (95% CI = 0.730–0.894, *P* < .001), 0.847 (95% CI = 0.775–0.919, *P* < .001), and 0.924 (95% CI = 0.863–0.984, *P* < .001). 2D-STI combined with stratified strain imaging can provide a more comprehensive, objective, and accurate assessment of myocardial synchronized exercise and clinical prognosis in patients with HFpEF, and LVGLS, sub-epicardium LS, and PSD values can be used in clinical practice as noninvasive, sensitive indicators for predicting the occurrence of MACE.

## 1. Introduction

Heart failure preserved ejection fraction (HFpEF) has a large clinical heterogeneity, clinical symptoms are mostly atypical, untimely treatment will lead to further deterioration, and the prognosis is unsatisfactory.^[1]^ It has been found^[2]^ that conventional ultrasound evaluation of left ventricular structure and pumping function has greater limitations in HFpEF, and that assessment of myocardial synchronized motion is essential for an in-depth understanding of the mechanisms of HFpEF and its clinical prognosis. Abnormal synchronized myocardial motion is closely associated with local myocardial ischemia, infarction, apoptosis, and fibrosis.^[3]^ Two-dimensional speckle tracking imaging (2D-STI) is a new imaging technique that has been studied in depth in clinical practice and is capable of tracking myocardial motion in real-time, independent of the angle of the ultrasound beam and with high sensitivity.^[4,5]^ Hierarchical strain imaging based on 2D-STI can further differentiate different layers and segments of the myocardium and provide more information about myocardial motion. Peak strain dispersion (PSD), transmural pressure difference, post systolic shortening (PSS), and early systolic lengthening can provide a more objective and accurate measure of LV myocardial motion incoordination. Exercise incoordination.^[6–8]^ Searching the literature, fewer studies are using 2D-STI and stratified strain imaging to assess myocardial synchronized motion in heart failure patients. Based on this, this study focuses on the assessment of myocardial synchronized motion and clinical prognosis in patients with HFpEF by 2D-STI stratified strain imaging to provide a reference basis for clinical practice.

## 2. Methods

### 2.1. General information

We retrospectively summarized 146 patients with HFpEF diagnosed in our hospital from January 2022 to January 2023 as the study subjects, including 86 males and 60 females, under an average age of (63.5 ± 4.9) years. Inclusion criteria:① aged between 18 and 75 years old; ② meet the diagnostic criteria of HFpEF, that is, heart failure symptoms + left ventricular ejection fraction (LVEF) ≥ 50% + plasma brain natriuretic peptide (BNP) ≥ 500 pg/mL; ③ complete 2D-STI and stratified strain imaging, with clear and preservable images; ④ comprehensive drug and rehabilitation therapy according to the recommendations of heart failure guidelines. Exclusion criteria: ① acute heart failure, heart failure with reduced ejection fraction, heart failure caused by congenital heart disease, cardiomyopathy, myocarditis, etc, heart failure secondary to liver cirrhosis and tumor chemotherapy; ② being under anti-heart failure treatment; ③ severe liver, kidney, and lung dysfunction.

Our study conformed to the principles outlined in the Declaration of Helsinki (Br Med J 1964; ii: 177), equipped with ethical approval [(2024) Ethics approval No. (01)], along with integral clinical and follow-up data.

### 2.2. Research methods

#### 2.2.1. Ultrasound

**Figure 1.**
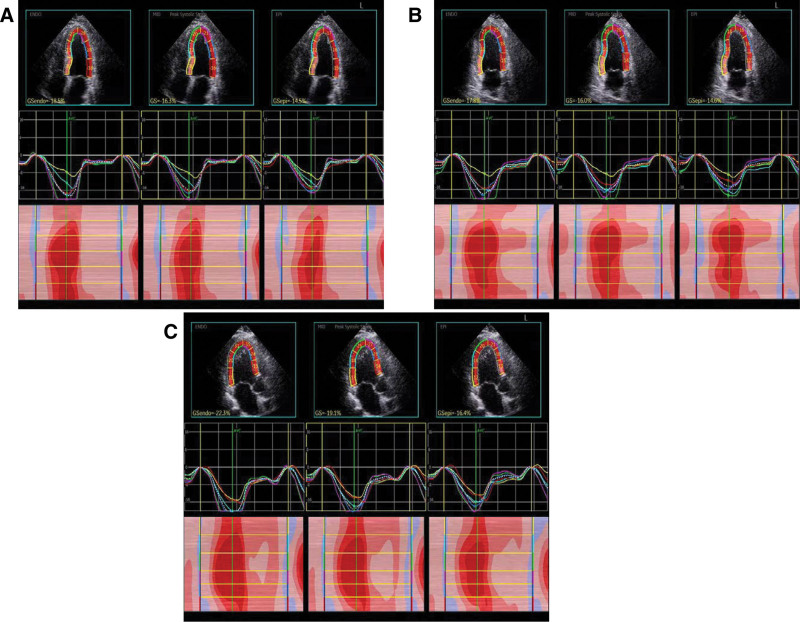
Two-dimensional longitudinal layered strain and strain curve of left ventricular myocardium. (A) sub-endocardium, mid-myocardium, and sub-epicardium GLS in 4-chamber heart section; (B) sub-endocardium, mid-myocardium, and sub-epicardium GLS in 2-chamber heart section; (C) sub-endocardium, mid-myocardium, and sub-epicardium GLS in 3-chamber heart section.

**Figure 2.**
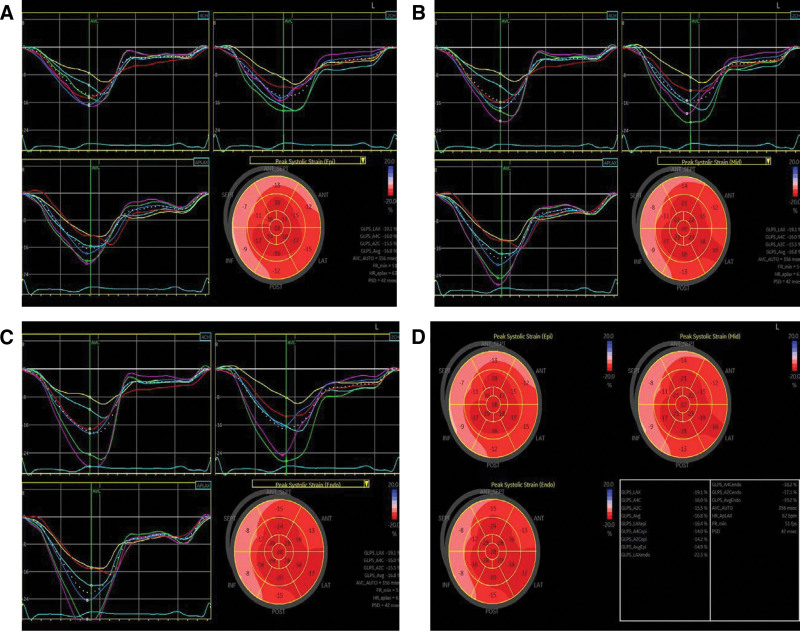
Bull’s eye view of longitudinal strain in myocardial segments of the left ventricle. (A) Bull’s eye view of longitudinal strain in sub-epicardium; (B) bull’s eye view of longitudinal strain in mid-myocardium; (C) bull’s eye view of longitudinal strain in sub-endocardium; (D) bull’s eye view collection.

We used the GE Vivid E95 ultrasonic diagnostic instrument with M5S probe, under a frequency of 1.5 to 4.6 MHz, which had an automatic functional imaging function and Echo PAC 203 image processing software. Patients were placed in the left lateral position, connected to the electrocardiogram, and the M5S probe was activated to routinely inspect all sections of the heart transthoracically to observe the morphology and structure of the heart, valvular activity, and hemodynamics, etc, then to capture and store 3 to 5 consecutive cardiac cycles, and the graphics of the apical 4-chamber, 2-chamber, and 3-chamber heart were frozen. Left ventricular end diastolic diameter and volume and left ventricular end systolic diameter and volume were measured by the biplane Simpson method, and LVEF values were calculated. Then the images were imported into the Echo PAC 203 workstation, and the stored dynamic apical 3-chamber, 4-chamber, and 2-chamber heart images were sequentially selected, and the Q-analysis mode speckle tracking analysis was entered, and the tracing point was placed at the mitral annulus and apical endocardium on both sides by clicking on 2D-strain, and the software automatically tracked the myocardial motion to generate the region of interest, and the edges of the endocardium and epicardium were manually adjusted so that the thickness of the myocardium was consistent, and then clicking on Approve, which was the first time in this study. The software automatically tracked the myocardial motion to generate the region of interest, manually adjusted the endocardial and epicardial edges to make them consistent with the thickness of the myocardium, and then clicked Approve to automatically generate the strain values of the 3 layers of the myocardium, the strain curve graphs, and the bull’s eye diagrams,^[9,10]^ and automatically calculated the left ventricular global longitudinal strain (LVGLS), the sub-endocardium, middle layer, and sub-epicardium LS, as well as the basal, intermediate, and apical LS. The standard deviation of the time to PSD; ΔLS is the difference between sub-endocardium and sub-epicardium myocardial LS. PSS is defined as the difference between peak negative strain in the cardiac cycle and peak strain in systole divided by the maximal strain amplitude in the cardiac cycle, and early systolic lengthening is defined as the ratio of the amplitude of the positive strain in systole to the maximal strain amplitude in systole, see Figures 1 and 2 for detailed information. The image acquisition and parameter measurements were performed solely by experienced attending and deputy chief physicians of the department of ultrasound who experienced in more than 5 years to ensure the stability of the results.

#### 2.2.2. Clinical information

Records include sex, age, body mass index, underlying diseases (hypertension and diabetes mellitus), history of myocardial infarction, history of stenting, mean percentage of coronary artery diameter stenosis, and plasma BNP levels before admission to the hospital for treatment.

#### 2.2.3. Clinical treatment and follow-up

After admission, the stenosis degree of target vessel diameter was evaluated, then appropriate treatment methods were selected, such as stent implantation or intensified drug therapy, including combination of angiotensin receptor enkephalinase inhibitors/angiotensin converting enzyme inhibitors + beta blockers + aldosterone receptor antagonists + sodium glucose cotransporter 2 inhibitors, while blood pressure, blood glucose, and blood lipids were actively controlled.

After 1 month of treatment, we measured the above ultrasound parameters again, and follow-up ended in January 2024, and the median follow-up time totaled 17 months. We recorded major adverse cardiac events (MACE), which mainly included heart failure exacerbation, rehospitalization, new infarction and stroke, malignant arrhythmia, and cardiac death. Then we divided 146 patients into adverse and better prognosis groups.

### 2.3. Statistical methods

We conducted data processing using SPSS 25.0 statistical software. For measurement information adhering to normal distribution, we represented it as mean ± standard deviation. The distinction between the 2 groups was executed through a respective samples *t* test. In instances where measurement information did not follow normal distribution, we defined it as median and quartile, employing the Mann–Whitney *U* test for group comparisons. Additionally, comparisons between pretreatment and posttreatment within the same group were conducted using either a paired *t* test or Mann–Whitney *U* test. Count data [cases (%)] were subjected to comparison using the χ^2^ test. To identify risk factors, multifactorial Cox regression analysis was employed, utilizing the stepwise regression approach. The area under the curve (AUC), sensitivity, and specificity were calculated from the receiver operating characteristic (ROC) curve of the subjects. *P* < .05 is considered a statistically significant difference.

## 3. Results

### 3.1. Comparison of general information of the 2 groups of patients

MACE occurred in a total of 25 of 146 patients (17.12%). Compared with the better group, patients in the adverse group had an increase in age, incidence of myocardial infarction, and elevated plasma BNP level before admission (*P* < .05), see Table 1 for detailed information.

**Table 1:** Comparison of general information of the 2 groups of patients.

Event	Poor group (n = 25)	Good group (n = 121)	t/χ^2^ value	*P*-value
Follow-up (months)	14.0 ± 3.0	16.0 ± 3.7	−2.542	.012
Sex (m/f)	15/10	71/50	0.015	.913
Age (years)	65.2 ± 5.4	60.0 ± 4.3	5.264	<.001
BMI (kg/m^2^)	23.5 ± 1.6	23.7 ± 1.8	−0.385	.701
Hypertension (cases [%])	10 (40.0)	55 (45.5)	0.250	.617
Diabetes mellitus (cases [%])	6 (24.0)	30 (24.8)	0.007	.933
Myocardial infarction (cases [%])	15 (60.0)	41 (33.9)	5.977	.014
Stent placement (cases [%])	19 (76.0)	79 (65.3)	1.077	.299
Mean percentage of coronary artery diameter stenosis (%)	85.9 ± 6.9	83.4 ± 6.6	1.749	.083
Pretreatment BNP (pg/mL)	742.3 ± 93.6	691.4 ± 91.0	2.530	.012

BMI = body mass index; BNP = brain natriuretic peptide.

### 3.2. Ultrasound parameters comparison between the 2 groups of patients

Before treatment, there were significant differences in LVGLS, sub-endocardium and sub-epicardium LS, PSD and PSS values between the 2 groups (*P* < .05). The LVGLS, sub-endocardium, mid-level and sub-epicardium LS, PSD and PSS values in the better group at 1-month follow-up were profoundly improved compared with the pretreatment time (*P* < .05), but the adverse group did not (*P* > .05). See Table 2 for detailed information.

**Table 2:** Comparison of ultrasound parameters between the 2 groups of patients.

Event	Poor group (n = 25)	Good group (n = 121)	Z/t value	*P*-value
Pretreatment				
LVEDd (mm)	48.2 ± 4.0	48.9 ± 4.4	−0.665	.507
LVESd (mm)	34.2 ± 2.7	34.7 ± 3.0	−0.753	.453
LVEDV (mL)	109.5 ± 21.2	113.5 ± 23.9	−0.773	.441
LVESV (mL)	48.4 ± 9.3	50.3 ± 10.5	−0.803	.423
LVEF (%)	57.0 (53.5, 58.0)	56.0 (54.0, 57.0)	−0.621	.534
LVGLS (%)	−15.8 ± 1.5	−17.8 ± 1.7	5.452	<.001
Sub-endocardium LS (%)	−16.9 ± 0.9	−18.8 ± 1.1	7.834	<.001
Mid-myocardium LS (%)	−16.9 (−17.4, −16.4)	−17.3 (−18.0, −16.2)	−1.483	.138
Sub-epicardium LS (%)	−14.8 (−15.8, −14.5)	−16.8 (−17.7, −15.9)	−5.459	<.001
Basal segment LS (%)	−15.8 ± 2.3	−16.6 ± 2.1	1.715	.088
Middle segment LS (%)	−16.7 ± 1.3	−17.2 ± 1.6	1.683	.095
Apical segment LS (%)	−17.4 ± 1.5	−18.2 ± 2.0	1.887	.061
PSD	48.0 (42.0, 52.5)	33.0 (29.0, 37.0)	−6.666	<.001
⊿LS (%)	−2.1 (−3.0, −1.1)	−2.2 (−3.2, −0.8)	−0.164	.870
PSS (%)	−12.8 (−14.3, −10.3)	−10.9 (−11.9, −10.2)	−2.792	.005
ESL (%)	6.9 ± 1.9	6.1 ± 2.0	1.837	.068
Posttreatment				
LVGLS (%)	−15.7 ± 1.4	−19.1 ± 1.6*	9.916	<.001
Sub-endocardium LS (%)	−17.1 ± 0.9	−19.5 ± 1.1*	9.972	<.001
Mid-myocardium LS (%)	−16.9 ± 0.8	−17.7 ± 1.1*	3.530	.001
Sub-epicardium LS (%)	−15.3 ± 1.1	−17.5 ± 1.2*	8.820	<.001
Basal segment LS (%)	−15.7 (−17.4, −14.9)	−16.6 (−18.4, −14.7)	−1.187	.235
Middle segment LS (%)	−16.7 ± 1.5	−17.2 ± 1.7	1.421	.157
Apical segment LS (%)	−17.5 ± 1.5	−18.0 ± 2.1	1.096	.275
PSD	46.0 (40.0, 56.0)	27.0 (23.5, 30.0)*	−7.413	<.001
⊿LS (%)	−1.9 ± 1.2	−1.9 ± 1.5	0.152	.879
PSS (%)	−10.2 (−13.5, −9.2)	−9.2 (−9.6, −8.8)*	−3.904	<.001
ESL (%)	6.2 ± 1.4	6.3 ± 2.0	−0.210	.834

ESL = early systolic lengthening; ⊿LS = transmural pressure difference; LVEDd = left ventricular end diastolic diameter; LVEDV = left ventricular end diastolic volume; LVEF = left ventricular ejection fraction; LVESd = left ventricular end systolic diameter; LVESV = left ventricular end systolic volume; LVGLS = left ventricular global longitudinal strain; PSD = peak strain dispersion; PSS = postsystolic shortening.

* Good group comparison between posttreatment and pretreatment, *P* < .05.

### 3.3. Analysis of risk factors for the occurrence of MACE

Multivariate Cox regression analysis showed that pretreatment LVGLS, sub-endocardium LS, and PSD values were independent risk factors predicting the occurrence of MACE in patients with HFpEF (*P* < .05). See Table 3 for detailed information.

**Table 3:** Cox regression analysis of risk factors for the development of MACE in patients screened for HFpEF.

Factor	β	SE	Wald	*P*-value	HR value	95% CI
Age	0.075	0.044	2.886	.089	1.078	0.989–1.176
MI	−0.973	0.629	2.397	.122	0.378	0.110–1.295
BNP	0.001	0.003	0.061	.804	1.001	0.996–1.006
LVGLS	0.309	0.145	4.564	.033	1.362	1.026–1.809
Sub-endocardium LS	0.258	0.221	1.369	.242	1.295	0.840–1.996
Sub-epicardium LS	0.512	0.228	5.052	.025	1.669	1.068–2.609
PSD	0.073	0.030	5.867	.015	1.075	1.014–1.140
PSS	0.000	0.111	0.000	.997	1.000	0.804–1.242

BNP = brain natriuretic peptide; HFpEF = heart failure preserved ejection fraction; LVGLS = left ventricular global longitudinal strain; MACE = major adverse cardiac; PSD = peak strain dispersion; PSS = postsystolic shortening.

### 3.4. Performance analysis for predicting MACE occurrence

**Figure 3.**
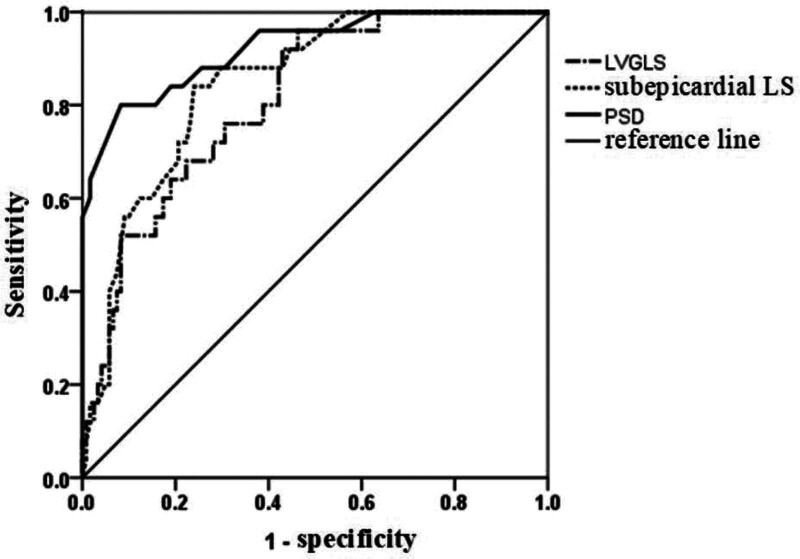
ROC analysis for predicting the occurrence of MACE. MACE = major adverse cardiac, ROC = receiver operating characteristic.

The ROC showed that the AUC of pretreatment LVGLS, sub-epicardium LS, and PSD values to predict the occurrence of MACE were respectively 0.812, 0.847, and 0.924, see Table 4 and Figure 3 for detailed information.

**Table 4:** Performance analysis of predicted MACE occurrence.

Norm	AUC	95% CI	*P*-value	Sensitivity (%)	Specificity (%)	Optimal threshold
LVGLS	0.812	0.730–0.894	<.001	76.6	68.3	−16.6%
Sub-epicardium LS	0.847	0.775–0.919	<.001	86.2	71.5	−15.6%
PSD	0.924	0.863–0.984	<.001	95.3	62.4	40.5

AUC = area under the curve; LVGLS = left ventricular global longitudinal strain; MACE = major adverse cardiac; PSD = peak strain dispersion.

## 4. Discussion

This study shows that the median MACE incidence about patients with HFpEF at 17 months of follow-up is 17.12%, which is in general agreement with previous reports.^[11,12]^ Patients in the adverse group have an increase in age, incidence of myocardial infarction, and elevated plasma BNP levels before admission. Advanced age, myocardial infarction, and BNP are considered traditional risk factors for heart failure prognosis. The adverse group shows elevated pretreatment LVGLS, sub-endocardium and sub-epicardium LS, as well as PSD values, while PSS values decrease. For patients with HFpEF under early LVEF still in the normal range and cardiac structure and function in the normal compensatory phase, the overall prognosis is favorable. However, 2D-STI combined with stratified strain imaging can detect myocardial synchronized dyskinesia early and more sensitively,^[13]^ such as sub-endocardium and sub-epicardium LS, PSD and PSS. LVGLS is the most widely used strain parameter for assessing myocardial motion and diastolic function and has important applications in several cardiac diseases, such as hypertensive heart disease, myocardial infarction, cardiotoxicity after oncology and chemotherapy and heart failure.^[14–16]^ However, since the heart consists of 3 layers of myocardium in different directions of motion, and the motion of each layer affects the overall myocardial motion synchronization, stratified strain imaging can further reflect the main components of myocardial synchronization disorders objectively, and provide richer information for diagnosis, development of more targeted interventions, and assessment of prognosis compared to conventional ultrasound imaging.^[17–19]^

This study shows that LVGLS, sub-endocardium, mid-myocardium, epicardium LS, PSD, and PSS values are significantly improved at 1-month follow-up compared to pretreatment in the better group, while the improvement in the adverse group is not evident. Orru D’Ávila LB et al^[20]^ enrolled a total of 2136 patients with heart failure (70.5% of them are with HFpEF) in 25 studies, measured peak oxygen consumption (VO_2_peak) and performed a Weber classification. The results indicate that the average LVEF and LVGLS of HFpEF patients in Weber A/B and C/D categories are similar (*P* > .05). However, low LVGLS values are correlated with decreased cardiorespiratory fitness in patients with HFrEF; the correlation between VO_2_peak and LVGLS is almost twice as high as that of LVEF, suggesting that LVGLS may be a better predictor of cardiorespiratory fitness in patients with HFrEF. Hu B et al^[21]^ conducted a prospective study on 92 patients with initial acute myocardial infarction. They performed echocardiography before coronary intervention, and during follow-up, 53 patients experienced cardiac events. The AUC for predicting cardiac events based on stratified strain imaging parameters is significantly higher than the conventional strain parameter LVGLS (*P <* .05). Therefore, it is concluded that stratified strain imaging is more valuable for the prediction of postinfarction cardiac risk and is closely related to the prognosis of patients with acute myocardial infarction during long-term follow-up. Coronary microvascular dysfunction (CMD) is associated with an increase in cardiovascular events in patients with nonobstructive coronary angina, especially heart failure, and traditional echocardiography struggles to identify early cardiac functional changes caused by CMD. Liu Q et al^[22]^ demonstrated that the difference in overall global waste work (GWW) and ΔPSD (before and after adenosine stimulation) in multivariate regression are independent risk factors for CMD. The ROC curve indicates that the composite predictive model comprising ΔGWW and ΔPSD has better diagnostic value for CMD (AUC = 0.913).

This study also identifies that pretreatment LVGLS (HR = 1.362), sub-epicardium LS (HR = 1.669), and PSD values (HR = 1.075) are crucial indicators for predicting MACE occurrence in patients with HFpEF. These indicators exhibit better performance in predicting MACE occurrence, demonstrating significant clinical application potential. However, the study has some limitations: firstly, it is a single-center, retrospective case summary with limited sample size and observation time, potentially affecting result stability. Secondly, the value of the selected LVGLS, sub-epicardium LS, and PSD values for predicting the prognosis of other types of heart failure patients requires further validation.

In conclusion, the combination of 2D-STI with stratified strain imaging provides a more comprehensive, objective, and accurate assessment of myocardial synchronized motion and clinical prognosis in patients with HFpEF. The values of LVGLS, sub-epicardium LS, and PSD can serve as noninvasive and sensitive indicators for predicting the occurrence of MACE in clinical practice.

## Acknowledgments

The authors acknowledge the financial support for Zhejiang Provincial Medical and Health Science and Technology Plan. The funders have no role in study design, data collection, analysis, interpretation, or manuscript writing.

## Author contributions

**Conceptualization:** Jingwan Chen, Xidan Wang, Yibo Zhou.

**Data curation:** Wenchao Weng.

**Formal analysis:** Jingwan Chen, Fuhua Chen.

**Investigation:** Fuhua Chen, Biao Tang.

**Methodology:** Fuhua Chen.

**Validation:** Xidan Wang.

**Writing – original draft:** Jingwan Chen, Wenchao Weng.

**Writing – review & editing:** Jingwan Chen, Yibo Zhou.
